# A qualitative process evaluation of a nasal spray intervention to prevent respiratory tract infections

**DOI:** 10.1371/journal.pone.0321314

**Published:** 2025-04-29

**Authors:** Amelia Dennis, Judith Joseph, Kate Greenwell, Sascha Miller, Jane Vennik, Laura Dennison, Sian Holt, Katherine Bradbury, Adam W. A. Geraghty, Paul Little, Lucy Yardley

**Affiliations:** 1 School of Psychology, University of Southampton, Southampton, United Kingdom; 2 Primary Care Research Centre, University of Southampton, Southampton, United Kingdom; University of Minnesota, UNITED STATES OF AMERICA

## Abstract

Nasal sprays could be used to prevent and manage respiratory tract infections (RTIs). As part of a randomized controlled trial (ISRCTN17936080), participants received one of two nasal sprays (gel-based vs. saline) and a digital intervention. The digital intervention used behaviour change theories to encourage nasal spray use to reduce the severity and occurrences of RTIs. We explored participants’ experiences of the digital intervention and nasal spray. We interviewed 31 participants (aged 19–80), sampled from the two nasal spray intervention trial arms across 3 winter seasons (including at the height of COVID-19). We analysed the interviews using thematic analysis and found two themes regarding facilitators and barriers to nasal spray use. The facilitators of nasal spray use revolved around belief in nasal spray efficacy for infection, belief the nasal spray is safe, motivation to avoid infection, sense of control over infection, and how the nasal spray is integrated into lifestyle. Barriers to nasal spray use included the belief the nasal spray is ineffective, belief the nasal spray is unnecessary, and usage difficulties. Overall, the results highlight the role of beliefs, lifestyle integration, and usage difficulties in nasal spray adherence, with implications for future digital interventions, such as addressing concerns about the nasal spray being perceived as medication.

## Introduction

Respiratory tract infections (RTIs) refer to a diverse group of infectious diseases affecting the upper or lower respiratory tract, encompassing conditions such as the common cold, influenza, and pneumonia. They are typically caused by viruses or bacteria and patients present with symptoms like coughing, sneezing, fever, and difficulty breathing. RTIs are very prevalent and have affect an estimated 17% of people monthly in the UK [1] and over 450 million global incidents in a year [2,3]. Typically, RTIs are self-limiting with mild to moderate symptoms [4,5]. However, among certain groups (e.g., older adults) it can lead to worse health outcomes such as severe illness and hospitalization [6]. In primary care, RTIs are one of the most frequent reasons for consultations and can add pressure to the healthcare system [7–9]. In turn, consultations on RTIs can lead to inappropriate antibiotic prescribing, that can fuel antibiotic resistance [10,11]. There is also the economic and well-being burden of RTIs, due to medical costs, missing work, and reduced health quality of life [6,12–14]. The COVID-19 pandemic further exacerbated the burden of RTIs on society [15] that led to millions of deaths [16] and millions having Long COVID which is associated with all ages [17]. Therefore, low-cost interventions are needed to aid the prevention of the RTIs and ease the long-term impact of RTIs.

The Immune Defence Study was a randomized controlled trial (RCT) that aimed to reduce the duration and incidence of RTIs through two different intervention approaches: modification of nasal environment (nasal sprays) and improvement of immune functioning (physical activity and stress management). In this qualitative process evaluation, we focus on the nasal spray interventions used to modify the nasal environment. There were two nasal spray interventions: a gel-based (antiviral) nasal spray and a saline nasal spray. Alongside the nasal sprays, a digital intervention in the form of a website (that included an instructional video) and paper booklet was provided to optimize nasal spray usage. The digital intervention was developed to promote nasal spray use through behaviour change mechanisms. Participants were invited to the Immune Defence RCT through their GP practice across three recruitment seasons: season one was December 2020 to April 2021; season two was September 2021 to April 2022; and season three was between September 2022 and April 2023. Therefore, recruitment seasons spanned different phases of COVID-19 including lockdown periods in the COVID-19 pandemic and non-COVID pandemic context (i.e., a normal winter for RTIs).

### Nasal sprays

Antiviral nasal sprays are designed to build a physical barrier that prevents the virus binding or entering the cell [18]. Research shows that, when used as early treatment for RTIs, antiviral nasal sprays (compared to a placebo) increase the recovery rate by reducing the duration of symptoms and viral load [19–22]. Saline nasal spray may aid the recovery of RTIs through irrigating the nasal passages and reducing infectious material from the sinuses [23], but recent evidence suggests that it is also highly likely that saline has an antiviral effect by supporting the production of substances that block virus replication [24,25]. A systematic review of saline nasal irrigation (nasal sprays and washes) in RTIs found some improvement in symptom control, however only five RCTs were identified and they were all evaluated as having a high risk of bias [26]. The Immune Defence trial demonstrated significant lower days of illness, sicks days from work, and courses of antibiotics in the gel-based and saline nasal spray group compared to the usual care group [27].

In the Immune Defence Study, the nasal spray arms of the trial were designed to assess the effectiveness of a gel-based nasal spray or a saline nasal spray on reducing RTIs, for intervention protocol see Vennik, Geraghty [28]. Participants were initially sent two bottles of the nasal spray, with further available on request to the trial team. The gel-based nasal spray was Vicks First Defence spray (Proctor and Gamble, Harrogate, UK) and the saline nasal spray was Sterinase (Earol, Glasgow, UK). Both the nasal sprays were concealed by removing the manufacturer labels and adding the study labels of either liquid-based (for saline) or gel-based (for Vicks First Defence). The two nasal sprays are available to purchase over-the-counter at supermarkets and pharmacies in the UK and are registered as medical devices.

### Digital intervention

**Fig 1:**
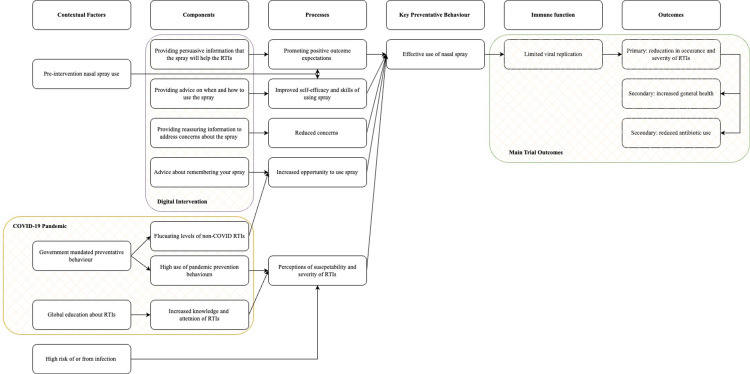
Logic model of behaviour change for the digital intervention. COVID-19 pandemic box refers to contexts arising from the pandemic that may influence nasal spray usage.

The digital intervention was designed to increase nasal spray usage behaviour through three key mechanisms drawn from behaviour change theories including social cognitive theory [29] and necessity-concerns framework [30,31]. See Fig 1 for the logic model and Williamson, Dennison [32] for intervention development.

We drew on social cognitive theory [29] to increase positive outcome expectancies and improve self-efficacy. The digital intervention was designed to promote positive outcome expectations by building confidence in the preventive benefits of nasal sprays (i.e., belief that the nasal spray can help prevent RTIs), emphasizing the sense of protection they may provide, and reinforcing the importance of maintaining other RTI prevention behaviours. To improve self-efficacy for nasal spray use, the digital intervention emphasised the simplicity of spray alongside clear instructions on how to use the nasal spray. Participants were instructed to use the nasal spray in three ways during the trial: at the first signs of infection, after potential exposure to infection (e.g., public transport, supermarkets, cafes/pubs), and after prolonged exposure to infection (e.g., living with or close contact with someone who has an infection), see Supplementary Materials for more details. The digital intervention also capitalized on the necessity-concerns framework [30,31] to reduce concerns of nasal sprays through providing reassuring information, such as misconceptions and apprehensions about overuse, side effects, and hygiene. Since the digital intervention was designed to guide nasal spray use through behaviour change techniques, our analysis of nasal spray usage includes an evaluation of how the digital intervention influenced participants’ belief and behaviour.

In the logic model, we also accounted environmental factors included in social cognitive theory [29]. First, we expected previous use of nasal sprays to impact people’s expectations of nasal spray efficacy and self-efficacy. For example, Williamson, Dennison [32] found people who had used nasal sprays before reported greater confidence in using nasal sprays. Second, we expected that people at high risk of and from infection would perceive greater susceptibility and severity from RTIs and therefore, use the nasal spray to mitigate this risk, in line with the health belief model [33]. Third, we accounted for the influence of the COVID-19 pandemic. The COVID-19 pandemic and lockdown may have reduced opportunities for participants to use the nasal spray, for example fewer non-COVID-19 RTIs during lockdown [34]. Additionally, it was expected the COVID-19 pandemic may have increased the public’s understanding of RTI transmission and attempts to prevent infection [35].

### The current study

In the current study, we present a qualitative process evaluation of the nasal spray interventions. We aimed to understand factors influencing nasal spray usage including both pre-existing individual contexts and factors from the digital intervention, which was developed to promote nasal spray use. Our primary research question was: ‘What factors and mechanisms underlie nasal spray usage?’

## Method

This was a qualitative process analysis study that is part of the Immune Defence RCT (28). Ethical approvals were granted by the National Research Ethics Committee, NHS, and the University of Southampton (REC: 20/SS/0102; IRAS: 288431; ERGO: 56474). Participants gave both online written and verbal informed consent to participate in the study before taking part. Participants were debriefed and received a £10 voucher to thank them for their participation. This study was written up in line with Standards for Reporting Qualitative Research checklist [36] (see Supplementary Materials).

### Participants

All participants who consented to take part in the Immune Defence study were also invited to give optional consent to be contacted for an interview. Participants in the two nasal spray arms (gel-based and saline sprays) who consented to be contacted were purposively sampled based on demographics and characteristics such as ethnicity, age, gender, socio-economic status, risk from infection, incidence of RTIs, and across the three recruitment seasons. Potential participants were invited by email or phone call at least three-months after first using the intervention. Thirty-one participants were interviewed, aged between 19 and 80 years old (see Table 1 for demographic information). There was a near equal split between the gel-based nasal spray (*n* = 16) and saline nasal spray (*n* = 15) arm (see Table 2 for participants by intervention). We finished recruitment when data saturation appeared to have been achieved while ensuring diversity across demographics and recruitment seasons, meaning no new information was obtained in the later interviews [37].

**Table 1:** Participant characteristics for antiviral and saline groups.

	Both sprays	Antiviral	Saline
**N participants**	31	16	15
**Recruitment Season N (%)**			
Season 1	13 (41.9)	9 (56.3)	4 (26.7)
Season 2	8 (25.8)	3 (18.8)	5 (33.3)
Season 3	10 (32.3)	4 (25.0)	6 (40.0)
**Age**			
Mean (SD)	57.07 (16.97)	57.38 (16.74)	56.73 (17.79)
**Gender N (%)**			
Female	14 (45.2)	7 (43.8)	7 (46.7)
Male	17 (54.8)	9 (56.3)	8 (53.3)
**Ethnicity N (%)**			
White - British	22 (71)	12 (75.0)	10 (66.7)
White - Other	1 (3.2)	0	1 (6.7)
Pakistani	1 (3.2)	1 (6.3)	0
Black African	1 (3.2)	0	1 (6.7)
Asian - Other	1 (3.2)	1 (6.3)	0
Indian	2 (6.5)	2 (12.5)	0
Irish	1 (3.2)	0	1 (6.7)
Any other mixed/multiple ethnicity	2 (6.5)	0	2 (13.3)
**Education N (%)**			
No formal	3 (9.7)	1 (6.3)	2 (13.3)
GCSE	5 (16.1)	0	5 (33.3)
HND	4 (12.9)	3 (18.8)	1 (6.7)
A level	5 (16.1)	4 (25.0)	1 (6.7)
Degree	6 (19.4)	5 (31.3)	1 (6.7)
Higher degree	0	0	0
Post-graduate	6 (19.4)	2 (12.6)	4 (26.6)
Other	2 (6.5)	1 (6.3)	1 (6.7)
**Deprivation**			
Mean (SD)	6.30 (2.42)	6.63 (2.50)	5.93 (2.37)
**Number of infections in the last 12 months**			
Mean (SD)	4.47(4.80)	6.33(5.68)	2.80(3.29)

**Table 2:** Participants allocation to the nasal spray interventions.

Intervention	Participant
Gel-based (antiviral) nasal spray (*n* = 16)	Participant 2, 4, 5, 6, 7, 8, 9, 10, 12, 15, 18, 19, 24, 27, 28, 30
Saline nasal spray (*n* = 15)	Participant 1, 3, 11, 13, 14, 16, 17, 20, 21, 22, 23, 25, 26, 29, 31

### Data collection

Data collection took place between 10^th^ May 2021 and 5^th^ May 2023 to capture participants in each of the three seasons of recruitment to the trial. Thirty participants were interviewed over the phone with interviews being audio-recorded. The recordings were then transcribed verbatim by a third-party company that removed any identifiable information. One participant was interviewed over email due to hearing impairments; while this adaptation was helpful for inclusivity, it limited the depth of responses, as the participant provided written answers to the main topic guide questions without follow-up prompts or probing. Interviews ranged between 26 and 80 minutes, lasting an average of 48 minutes. The interviews were conducted by KG, SM, SH, LD, and JJ, who are all experienced qualitative researchers and some of whom (KG, SH, LD) had developed the digital intervention. To mitigate potential bias, KG, SH, and LD maintained reflective notes after each interview, while data analysis was conducted by an independent researcher (AD) to ensure objective interpretation of the findings. Participants completed an online form to give consent before the interviews.

A semi-structured interview schedule (see Supplementary Materials) was developed based on the behaviour change mechanisms that guided the intervention’s development (see Fig 1). The interview guide explored four key themes. First, any previous RTIs they had before starting the trial (e.g., “Can you tell me about any infections that you tend to have”). Second, their beliefs and experiences of using the nasal spray such as the situations they used the nasal spray, why they used the spray in these situations, and changes since using the spray (e.g., “Can you tell me what stopped you from using the spray?”). Third, their experiences of the digital intervention including the amount they used the digital intervention website and if it helped them use the nasal spray (e.g., “What was it about these resources [the website and booklet] that helped you to use the nasal spray?”). Fourth, to account for the impact of COVID-19 through exploring how COVID-19 influenced infection prevention attitudes and behaviours (e.g., “How have you found using the nasal spray alongside these things [COVID-19 pandemic]?”). Open-ended questions allowed participants to describe their views and experiences in their own words and the opportunity to focus on what was most important to them.

### Patient and public involvement

Patient and Public Involvement (PPI) panel have contributed to the development of the study and study materials, some from the grant application stage. The PPI panel had experience of reoccurring RTIs and/or health conditions placing them at high risk of or from infections. The contributions from the PPI panel included editing and reviewing the logic model, intervention development, participant information sheets, consent forms, and interview schedules as well as piloting nasal sprays and interviews. Specifically, PPI panels members reviewed the interview schedule leading to the interview schedule being simplified and shortened.

### Data analysis

A thematic analysis was conducted from a critical realist perspective [38,39]. The analysis was conducted using NVivo V.14 by AD and JJ, who were both unaware of whether participants were allocated to the antiviral or saline nasal spray intervention. We also did not look for any differences between the two nasal sprays as we were interested in what prompted use of the nasal spray. AD and JJ are both psychology researchers with previous experience of conducting qualitative analysis. The first stage included a thematic analysis, following familiarization of the transcripts, AD developed descriptive codes from inductively coding a selection of transcripts and an initial coding manual was developed. AD and JJ then developed and refined the coding manual while analysing the rest of the transcripts. A data-led approach was taken when developing the themes. The themes and sub-themes were then continually examined and refined, and any discrepancies and inconsistencies were discussed. Then, AD conducted an abductive analysis to move beyond purely descriptive themes towards exploring mechanisms underlying nasal spray use. This process involved exploring the themes and sub-themes to infer the mechanisms underlying nasal spray behaviour and the context these mechanisms occurred in. This resulted in context-mechanism-outcome (CMO) configurations that included configurations of moderators (individual beliefs and personal contexts influencing adherence) and mediators (modifiable attitudes, experiences, and skills) on outcomes (nasal spray behaviour). The final stage involved a retroductive analysis, in which a logic model was used to abstract the findings into generalizable principles, ensuring their applicability to future interventions. The COVID-19 pandemic was taken into account as a factor influencing moderators and mediators through an inductive, data-led approach. Throughout all stages of the analysis, the team (LY, AW, AG, SM, and KG) provided input into the themes in terms of interpretation of the data and coherence.

## Results

Two themes were developed from the data: facilitators of nasal spray use and barriers to nasal spray use.

### Facilitators of nasal spray use

**Fig 2:**
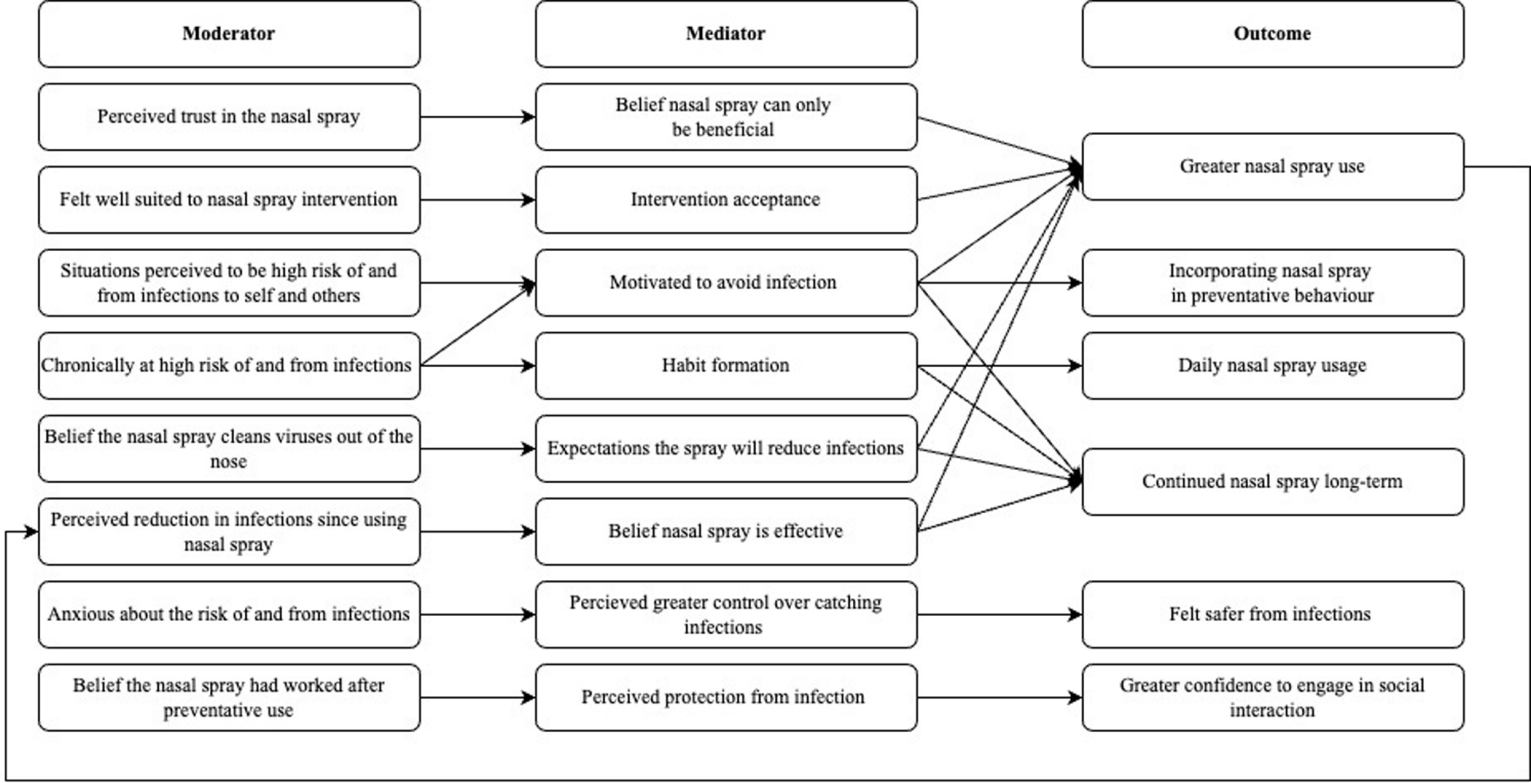
Logic model describing hypothesized links between context, mechanisms and outcomes in relation to facilitators of nasal spray use behaviour.

Facilitators of nasal spray use revolved around the five sub-themes of belief in nasal spray efficacy for infection, belief the nasal spray is safe, motivation to avoid infection, sense of control over infection, and nasal spray easily integrated into lifestyle. See Fig 2 for the logic model for facilitators of nasal spray use.

#### Belief in nasal spray efficacy for infection.

When participants wanted to thoroughly understand the nasal spray, they reported reading the digital intervention to aid understanding: *“I did. I looked at the booklet first and then went on to the website, so that was just to aid my understanding”* (Participant 23). Of the participants who read the instructions, some believed that the nasal spray worked by cleaning viruses out of the nose that was accompanied by an expectation that the nasal spray would help reduce their infections (as detailed in the instructions): “*if it [nasal spray] coats the little hairs in the start of the nasal passage and the germs get trapped in that and they’re killed, then hopefully I won’t get a chest infection”* (Participant 15). In this case, participants reported nasal spray use and a desire to continue using the nasal spray after the intervention finishes: *“use the spray to minimise or limit the amount of viral load in your nose. Which can potentially help with an ongoing illness, as well as essentially help prevent an illness. […] That’s one of the reasons why I said I would potentially continue with this beyond the study period.”* (Participant 24).

CMO configuration: When participants read the digital intervention and believed the nasal spray cleans the virus out of nose (context), they expected the nasal spray to reduce their infections (mechanism) resulting in greater nasal spray usage and a desire to continue using nasal spray long-term (outcomes).

If participants then perceived a reduction in their infections following nasal spray use, they believed the nasal spray was effective at reducing infection: *“it [nasal spray] has helped, I believe, reduce the infections”* (Participant 7), and contained medication: *“things that just help fight bacterial infections, help stave off infections […] I’d say either vitamins or medicines”* (Participant 3). These participants reported continued nasal spray usage: *“As the study went on, I just carried on using it because I was getting benefits”* (Participant 30) and a desire to continue using the nasal spray after the intervention finishes: *“I’d very much like to continue with that. It has actually, I believe, helped”* (Participant 7).

CMO configuration: When participants noticed a reduction in infections since using nasal spray (context), they believed the nasal spray was effective at reducing infection (mechanism) that reinforced nasal spray use and a desire to continue using nasal spray long-term (outcomes).

#### Belief the nasal spray is safe.

Some participants reported trusting that the nasal spray was safe: *“there’s not going to be anything in there that’s going to do me any damage”* (Participant 14). When participants had trust in the nasal spray, they felt they might as well use the nasal spray as it could only have benefits and that the worst that could happen was that it would not be effective at reducing infections as it would not be harmful: *“it’s only going to be positive, so even if it’s a placebo, it’s not going to mean I get anything worse […] it’s not going to be any harm”* (Participant 2). These participants expressed using the nasal spray: *“I just didn’t see any downside […] I just thought I might as well just try it”* (Participant 20).

CMO configuration: When participants trusted the nasal spray was safe (context), they believed the nasal spray could only be beneficial (mechanism) and reported greater nasal spray usage (outcome).

#### Motivation to avoid infection.

Some participants perceived themselves to be chronically at high risk of or from infections, typically due to health conditions. These participants were motivated to use the nasal spray as a way to avoid infection: “*I’m on immune suppressants, so I’m susceptible to picking up things anyway. So my thought is, it may add to my defence that way”* (Participant 4). These participants reported greater nasal spray use including using the nasal spray for infection management: *“if you feel you’ve got a cold coming on, you squirt it, great”* (Participant 26), and infection prevention: “*It’s given me something else to help me, I believe, avoid becoming infected.”* (Participant 7). These participants who felt at high risk of or from infection and used the nasal spray to avoid infection, viewed the nasal spray as part of their preventative behaviours: “*It’s given me something else that could contribute to keeping me, I won’t say safe, but healthier, less likely to become infected*” (Participant 7).

CMO configuration: When participants perceived themselves chronically at high risk of and from infection (context), they were motivated to use the spray to avoid infection (mechanism) leading to greater nasal spray usage, including nasal spray in preventative, and a desire to continue using nasal spray long-term (outcomes).

Participants also felt motivated to use the nasal spray in situations they perceived greater risk of or from infection to either themselves or others: “*whenever I feel I’m not safe, I will use that”* (Participant 27). These situations included when participants’ household members showed signs of infection, during the height of the COVID-19 pandemic, or when they were meeting someone who was medically vulnerable: *“I was so scared because I don’t want to have COVID”* (Participant 21). Some of these situations were identified in the instructions as contexts in which the nasal spray should be used (e.g., exposure to infection) but some were not (e.g., contact with someone medically vulnerable). In these situations, they used the nasal spray to avoid infection: *“I think I need it more, so I should be using it more than ever, because of the pandemic. I felt it will help me prevent this COVID that I’m avoiding”* (Participant 21). In this context, nasal spray usage only extended to the perceived high risk situation and when they no longer felt at risk they did not use the nasal spray as much: *“I haven’t really been thinking about it at all and I haven’t used it. I haven’t been thinking I’ll get COVID recently”* (Participant 29). If participants felt at high risk of infection (both chronically or situationally), they expressed intentions to continue using the nasal spray after the intervention ended to avoid infection: *“If I still had some I would just continue to it, in the hope that it would stop me getting any other infection”* (Participant 9).

CMO configuration: When participants were in situations that were perceived to be high risk of and from infection towards self and others (context), they were motivated to use the spray to avoid infection (mechanism) resulting in using the nasal spray in these situations and a desire to continue using nasal spray long-term (outcome).

#### Nasal spray easily integrated into lifestyle.

Participants who perceived themselves chronically at high risk of and from infection and used the spray to avoid infection formed a habit of using the nasal spray so they would not forget to use it: “*I thought I’m not going to remember to do different if I’ve been out, so I just sort of - it just naturally fell there [into a routine]”* (Participant 23). They formed habits by leaving the nasal spray in prominent places so they would remember to use the nasal spray: *“One is upstairs by the side of the bed […] and then I’ve got one at the side of the sofa”* (Participant 23) and by linking it to other habits: *“First thing in the morning I apply the spray. When I clean my teeth.”* (Participant 7). Participants who formed a habit of using the spray showed daily nasal spray usage: *“I was using it daily”* (Participant 17) and willingness to use the nasal spray after the intervention ends: *“I have been using the spray as soon as it started and I would say that I’d very much like to continue with that”* (Participant 7).

CMO configuration: When participants perceived themselves chronically at high risk of and from infection (context), they formed a habit to not forget to use the nasal spray (mechanism), resulting in daily nasal spray usage (outcome).

Some participants felt they were well suited to the nasal spray and were happy at their allocation to the nasal spray intervention. Participants felt this as they found the nasal spray easy to use: *“It was just easy to do”* (Participant 16), were used to using nasal sprays: *“I’m quite au fait with using a nasal spray”* (Participant 10), or they did not want to be in the lifestyle intervention: *“Diet and advice doesn’t suit everybody. I don’t think it would suit somebody like me”* (Participant 18). These participants expressed accepting the intervention through being happy at their intervention allocation: *“I was quite happy when I found out about it [allocation to the nasal spray intervention]”* (Participant 1) or positive expectations of using the nasal spray: *“I’ve taken nasal sprays lots of times in the past, so it’s a nasal spray […] an easy thing to do”* (Participant 14) and how they had used the nasal spray: “*I was really happy when I got put in the nasal spray group because I thought, I’m going to work with this, and I did. I’ve used it for when I’ve been poorly*” (Participant 18).

CMO configuration: When participants felt they were suited to the nasal spray intervention (context), they accepted the intervention (mechanism), resulting in greater nasal spray usage (outcome).

#### Sense of control over infection.

If participants were anxious about the risk of and from infections, it was reported the nasal spray provided them with a sense of control about contracting an infection as they were taking preventative action: “*I like feeling like I can do something; not just completely hopeless”* (Participant 12). This allowed participants to feel safer and less anxious about infections: *“it [nasal spray] has maybe made me worry less about COVID, or viral infections”* (participant 16);*“I’m certainly less anxious about them [infections] and that has been a massive thing […] having that anxiety lessened is a really, really lovely feeling”* (Participant 14).

CMO configuration: When participants were anxious about the risk of and from infections (context), the nasal spray gave them a sense of greater control over catching infections (mechanism), that resulted in them feeling safer from infections (outcome).

Additionally, if participants had used the nasal spray preventatively and felt it effective at preventing infection, they expressed feeling safer from infections: *“it gives you a more safe feeling”* (Participant 13), and a desire to engage in more social interaction: *“whole mental aspect of not mixing and of being afraid to see people was really tough. So I guess it [the nasal spray] gave me more confidence to start doing that again”* (Participant 14).

CMO configuration: When participants used the nasal spray and believe it worked after preventative use (context), they felt protected from infection (mechanism), leading to greater confidence to engage in social interaction (outcome).

### Barriers to nasal spray use

**Fig 3:**
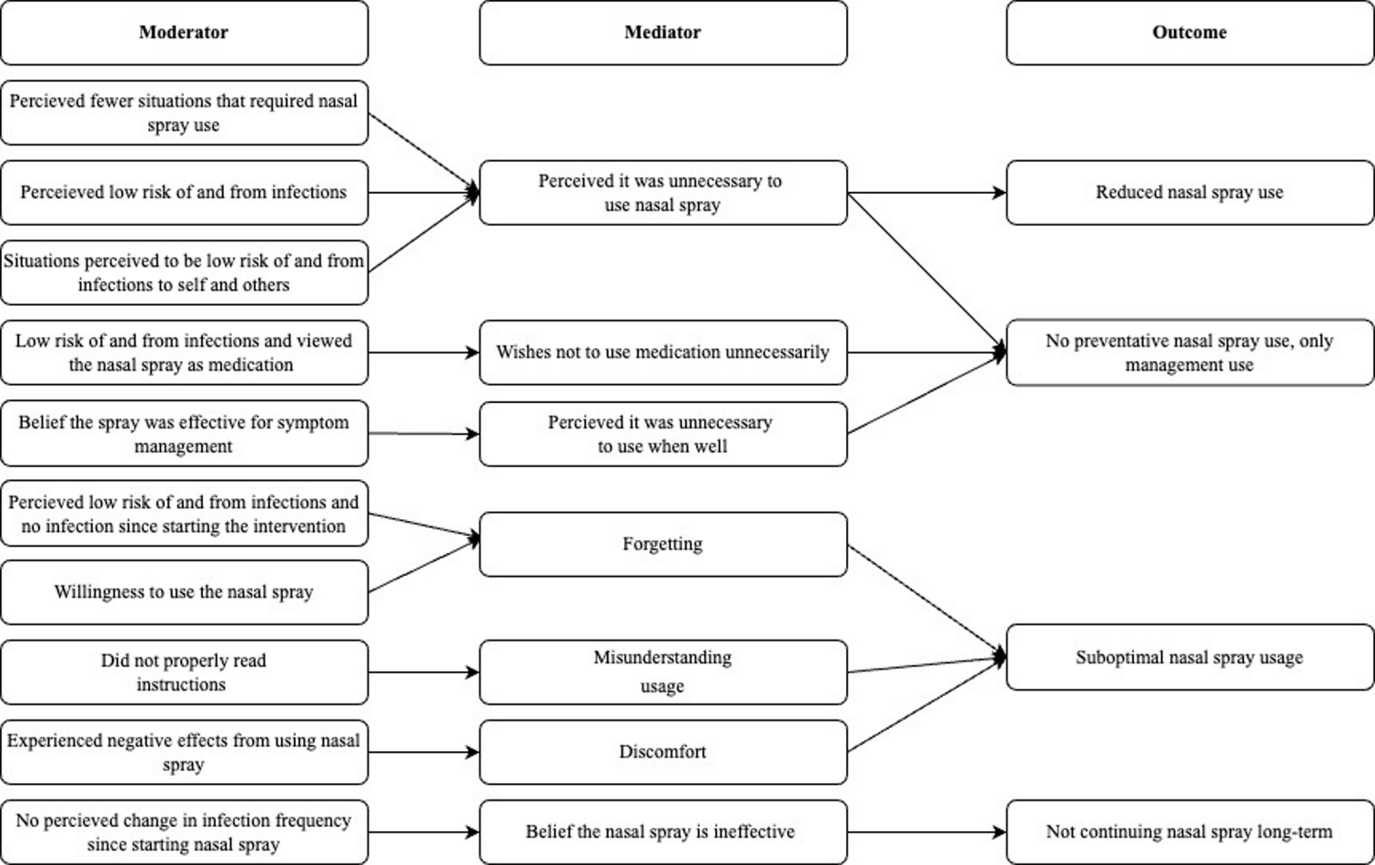
Logic model describing hypothesized links between context, mechanisms and outcomes in relation to barriers of nasal spray use behaviour.

Barriers to nasal spray use revolved around the three sub-themes of belief the nasal spray is ineffective, belief the nasal spray is unnecessary, and understanding usage difficulties. See Fig 3 for the logic model for barriers to nasal spray use.

#### Belief the nasal spray is ineffective.

If participants perceived no change in their infections since using the nasal spray, they believed the nasal spray was ineffective and could be a placebo: *“Is it normal saline water, because there was no effect, at all?”* (Participant 17). Most participants who believed the nasal spray was saline or a placebo were in the saline nasal spray arm: *“Well, I think it’s just a salt solution”* (Participant 13); *“just saline or something”* (Participant 20). However, some participants in gel-based arm also believed the nasal spray may have contained saline but this tended to be alongside other suggestions: *“it is an antibiotic or antiviral or just a normal saline”* (Participant 27); *“Not sure if it was the real stuff or a placebo”* (Participant 28). These participants reported not wanting to use the spray after the study: *“I doubt it [use nasal spray after intervention ends], to be honest”* (Participant 22) or that using it after the intervention would be based on the findings of the study: *“I suppose it depends on the results of the research”* (Participant 29).

CMO configuration: When participants perceived no change in their infections since using the nasal spray (context), they believed the nasal spray was ineffective or placebo (mechanism), resulting in less desire to continue usage long-term (outcome).

Belief the spray was effective also acted as a barrier to nasal spray use. Some participants had perceived the nasal spray to be effective for symptom management: *“If I do get anything [cold] normally the first thing I go for because it [nasal spray] seems to work quite quickly and quite effective”* (Participant 6) These participants did not see the point in using the nasal spray to prevent illness and only used it for symptom management: *“only [uses nasal spray] when I can feel one [cold] coming. If I feel anything, it might be a bit tingly or something. I don’t use it just for the sake of it, no”* (Participant 26).

CMO configuration: When participants perceived the nasal spray to be effective for symptom management (context), they did not see point in taking it when they were not ill (mechanism), then they only used it for symptom management and not preventatively (outcome).

#### Belief the nasal spray is unnecessary.

Some participants perceived that they had been in fewer situations that required using the nasal spray and believed that as they had not been in these situations the nasal spray was unnecessary to use and reported less nasal spray use. One instance was when participants had not been ill since starting the intervention and therefore reported little nasal spray usage for infection management: “*Haven’t felt the need to use it. Haven’t had any sniffles or anything*” (Participant 5). Due to the restrictions brought on by the COVID-19 pandemic, some participants had been in fewer situations in which they had contact with people who could have an infection; it was then perceived to be unnecessary to use the nasal spray preventatively: *“it says, you’re going to go out with lots of people to put this thing up your nose, but I haven’t done that yet”* (Participant 1). Nevertheless, some of these participants reported wanting to use the nasal spray for infection prevention or management if the situation arose: *“Oh, certainly if I’d got a cold. Anything that could help me with a stuffed up nose and a sore throat I would most certainly take”* (Participant 11).

CMO configuration: When participants had been in fewer situations that required nasal spray (context), felt it was unnecessary to use nasal spray (mechanism), leading to reduced nasal spray use (outcome).

Additionally, when participants perceived low risk of and from infection, they did not use the nasal spray preventatively because they felt it was not necessary: *“No, I wasn’t going to do that [use the spray preventatively] […] I don’t see myself as being in danger”* (Participant 22). Instead, these participants placed greater emphasis on using the nasal spray to manage symptoms: *“I had one or two days of a sore throat or a sniffle or whatever, and I’d use it at the beginning of that”* (Participant 29).

CMO configuration: When participants perceived themselves at low risk of and from infections (context), they felt it was unnecessary to use nasal spray (mechanism), resulting in only using the nasal spray for symptom management and not preventatively (outcome).

There were also specific situations in which participants perceived low risk of infection, they felt it was unnecessary to use the nasal spray and reported less nasal spray use. One situation in which participants perceived low risk was when they and those around them had taken preventative behaviour against COVID-19, such as vaccinations and social distancing. These individuals felt there was no need to use the spray as they had already mitigated their risk of infection: *“everybody’s wearing masks, aren’t they, at the minute. We’ve cut down our risk in that way. I wouldn’t have used in school I don’t think”* (Participant 5). Another situation in which participants perceived low risk of infection believed it was not necessary to use to spray preventatively was when they were around family and friends (who they did not live with). Participants suggested their family and friends did not pose a risk because “*they’ve all taken precautions through the pandemic*” (Participant 9) and *“are extremely healthy”* (Participant 11). This perception persisted even when their friends and family showed signs of infection: *“My grandson was here this weekend, and he’d got a bit of a throat infection, but that didn’t really prompt me to use the spray […] I don’t know whether it’s because it was my seven-year-old grandson I didn’t worry about it as much”* (Participant 31).

CMO configuration: When participants were in situation perceived to be low risk of infection (context), they felt it was unnecessary to use nasal spray (mechanism), resulting in reduced nasal spray use (outcome).

If participants perceived they were at low risk of and from infections and viewed the nasal spray as medication, they perceived using the nasal spray as unnecessary as they were well, and the nasal spray was not used preventatively: “*I don’t see the point of using it if I’m feeling fine”* (Participant 26). There was also a belief that while the nasal spray may not be damaging to use preventatively there would be no benefit to using it preventatively: *“I imagine the ingredients aren’t particularly harmful, but still a lot of stuff to put in from a preventative point of view, so yes, for those reasons.”* (Participant 10) and that medication was not something to take when you are well: *“I did resist [using the nasal spray] because it is medicine and nothing to play with”* (Participant 11). However, these participants reported they would use the nasal spray for symptom management if they had a cold: *“if this nasal spray could ease my cold, my snuffed nose and such like, I would most certainly take it. It’s just I don’t take anything unnecessary, medicine”* (Participant 11).

CMO configuration: When participants perceived themselves at low risk of and from infection and viewed the nasal spray as medication (context), and they did not like using medication unnecessarily (mechanism), resulting in only using the nasal spray for symptom management and not preventatively (outcome).

#### Understanding usage difficulties.

Some participants reported that the discomfort from using the nasal spray was a barrier to nasal spray usage. Some participants reported negative effects from using the nasal spray. This included side effects like nose bleeds: *“I was getting a few more nosebleeds than I used to get”* (Participant 2) and general dislike of using the nasal spray: *“No, not really. It is rather unpleasant sticking things up your nose”* (Participant 22). Some people reported physical or psychosocial discomfort: *“you get that horrible sensation at the back of your throat”* (Participant 18) *“It will make it dry my nose and I need to pick it. That make me sometimes feel awkward or embarrassing”* (Participant 27). These participants who had experienced negative effects reported suboptimal nasal spray usage such as only using the nasal spray in one nostril: *“If it was my right nostril, I would not be using the spray, because that actually irritated it”* (Participant 7) and reduced nasal spray usage: “*I have stopped it for two months, purely because of the severity that I got a post-nasal [drip]”* (Participant 17).

CMO configuration: When participants experienced negative effects from using the nasal spray (context), they felt discomfort (mechanism), leading to suboptimal nasal spray usage (outcome).

If participants perceived themselves to be at low risk of and from infections and had not contracted an infection since starting the intervention, they may have forgotten about the using nasal spray, and expressed suboptimal nasal spray use: “*I’ve probably forgotten about it […] I haven’t used it. I haven’t really felt coldy, but it’s sort of gone out of my mind a bit more”* (Participant 29). Some of these participants also forgot about the situations that the nasal spray could be used: “*You said about my family, and I said oh, I forgot about that situation as well, when you could use it”* (Participant 2). Some of these participants made suggestions for future interventions to include reminders or prompts about the nasal spray so participants would remember to use it. Suggestions included texts prompts, email reminders, or prompts to put around your house: *“A little mini poster, small poster that you could stick up on your fridge”* (Participant 29).

CMO configuration: When participants who perceived themselves to be at low risk of and from infections and had not contracted an infection since starting the intervention (context), they forgot about using and situations for the nasal spray (mechanism), resulting in suboptimal nasal spray usage (outcome).

Additionally, even when willing to use the nasal spray, some participants would forget to use the nasal spray in preventative situations, despite intending to: “*we didn’t always remember to do it [use the nasal spray]*” (Participant 16) and reported inconsistent nasal spray usage in preventative situations: *“if I go shopping, I don’t always use it when I get back. I don’t always remember frankly*” (Participant 31).

CMO configuration: If participants were willing to use the nasal spray (context) but forgot to use nasal spray in preventative situations (mechanism), it led to suboptimal nasal spray usage (outcome).

Some participants reported not properly reading the nasal spray instructions: *“I didn’t read what I was given”* (Participant 3) because they had used nasal sprays before or believed they knew how to use them or felt they understood how to use the nasal spray after reading through the instructions once: *“Well, I think I had no need to [go back to instructions] because I had all the information that I needed”* (Participant 22). Some of these participants misunderstood when the nasal spray should be used and reported suboptimal nasal spray usage. For example, many participants reported thinking the spray should be used four times a day: *“I always try and do it four times a day, as the instructions said”* (Participant 3). One participant thought the spray was not intended to be used preventatively: *“the spray wasn’t actually intended to be a preventative measure anyway so that’s why I would not actually take it”* (Participant 19) and another participant thought you could only get two bottles of the nasal spray and thus had not used it for a while: *“I haven’t had any for months. As I say, I just used the initial two. I thought that was the test”* (Participant 9). This illustrates that some participants did not read the instructions fully as ordering extra nasal sprays was included in the instructions which other participants had acted on: *“Whoever I’ve contacted for new bottles has always sent me an email saying, ‘Yes, no problem,’ because I did that by email rather than phoning up. […] It’s in the booklet, it’s on the web; when you run out just ask for more”* (Participant 7).

CMO configuration: If participants had not properly read the instructions (context), they misunderstood intended usage (mechanism), resulting in suboptimal nasal spray usage (outcome).

## Discussion

In this study, we aimed to understand the factors (pre-existing individual contexts or the digital intervention) that influenced nasal spray usage, with nasal spray usage supported with a digital intervention. The results from this study highlight the barriers and facilitators of nasal spray use. The facilitators of nasal spray use include believing the spray was safe and effective, motivation to avoid infection, and easily integrated in lifestyle that provided participants a sense of control over infection. Barriers to nasal spray use centred around the belief the nasal spray is ineffective or unnecessary and usage difficulties (e.g., misunderstanding instructions and discomfort).

The digital intervention targeted three key behaviour change mechanisms. First, increasing positive outcome expectancies of the nasal spray. In line with social cognitive theory [29], we found that positive expectations facilitated nasal spray use, particularly trust that the spray was safe and could only be beneficial and an expectation that the nasal spray would work through modifying the nasal environment. However, ironically, the perceived efficacy of the sprays when used at the early signs of infection were also a barrier to preventative use, as this increased confidence in being able to mitigate the effects of infection and reduced the relevancy of using the nasal spray preventatively when they were not ill. Further development work with users is required to inform any future implementation of preventative spray use, to help identify circumstances in which this is acceptable and feasible (for example, in situations of unusually high risk to self or others).

The second mechanism focused on increasing self-efficacy for nasal spray use. We found that being motivated to avoid infection, habit formation, thinking the nasal spray was easy to use, and being used to using nasal sprays, all facilitated nasal spray use, whereas forgetting and misunderstanding instructions were barriers to nasal spray use. This highlights the role of self-efficacy related to the action of using the nasal spray use and learning about the nasal spray, both of which have been associated with medication adherence [40,41].

The last mechanism worked to reduce concerns of nasal sprays. Despite our best efforts to position using sprays as analogous to hand hygiene rather than medication use, concerns about over-medication remained a barrier to preventative use, particularly when accompanied by a belief that preventative use was unnecessary. It is common for people to report little necessity and high concerns for over-the-counter medication [42], which can lead to less medication adherence [43] in line with the necessity-concerns framework [30,31]. However, the belief the nasal spray was medicine reinforced usage when people felt the nasal spray was effective. Our findings indicate barriers to nasal spray use such as concerns about over-medication, forgetting, and preventing nasal pain (by correct spray technique) remain and should be addressed by future research.

Most participants were enthusiastic about using the spray to avoid or reduce infection when they were symptomatic or when they perceived themselves or people they were meeting to be at high risk of or from infection, which are the most important contexts for preventing illness. This is in line with the health belief model [33] that suggests that greater perceived susceptibility and severity of an illness will lead people to take action to mitigate risk [44]. Research from previous studies and the COVID-19 pandemic shows that higher perceived risk predicts greater engagement in preventative behaviour (e.g., hand washing) [45–47]. Therefore, the nasal spray could be used in future respiratory virus pandemics where people may perceive high risk but also in other situations where people are at high risk such as weakened immune systems, e.g., diabetes or kidney disease [48,49]. Additionally, public health campaigns can leverage these findings by explaining which situations increase susceptibility to infection and highlighting the benefits of using nasal sprays to prevent RTIs in these contexts. Also engaging in the preventative behaviour through nasal spray use can leave participants feeling at reduced perceived risk and more confident to engage in social interaction. Although this may lead to increased risk of contracting an infection [50], social isolation negatively impacts mental health [51,52] and engaging in social interaction while taking preventative behaviour (such as using a nasal spray) may have mental health benefits that outweigh infection risk. Therefore, nasal spray usage may have secondary benefits that include improved mental health by alleviating some of the anxiety around contracting an RTI. We also found that people were often reluctant to use the spray preventatively, especially in situations they considered low risk, such as meeting family, friends, or familiar coworkers. This is in line with research during the COVID-19 pandemic that shows when interacting with friends (vs. strangers) people perceived less risk and engaged in less preventative behaviours [53]. Side effects and discomfort of nasal spray use was also a barrier. The main trial identified that there were more adverse effects from the gel-based nasal spray compared to the saline nasal spray [27]. Given that both nasal sprays group reduced days off illness, sick days from work, and antibiotic courses in comparison to the usual care group [27], it may be that the saline nasal spray has more behavioural benefits given fewer adverse side effects.

This study has several limitations. First, the advertised was advertised among people at high risk of or from a RTI and people who volunteered to be in the trial (that was advertised as a nasal spray intervention for RTIs) are likely to be more highly motivated to use the nasal spray and reduce RTIs than the average person. Future studies could aim to recruit people at low risk of or from RTIs to better assess the intervention’s effectiveness across different risk groups and aid infection prevention in everyday settings. Second, the trial began in December 2020 with 41.9% of participants in this study starting the trial and being interviewed during the height of the COVID-19 pandemic. There was public adherence to preventative behaviours [54], as a result it may have increased willingness to participate and use the nasal spray, therefore, future studies could conducting trials in a non-pandemic context. A strength of this study, is that the demographics of the sample corresponds well to the demographics of the 2021 census [55] with good representation of age, gender, education levels, and ethnic minorities suggesting generalizability of findings to the general population as well as specific populations we were able to capture (e.g., those with weakened immune systems). Last, this process evaluation was part of the Immune Defence RCT and findings may be partly specific to the trial context since participants’ nasal spray use was tied to their experience of the trial itself. As a result, this qualitative analysis does not capture nasal spray usage behaviours in a real-world setting and future research could explore adherence and attitudes outside of a trial framework.

### Conclusion

In this study, we aimed to identify factors that influenced the use of nasal sprays following a digital intervention on reducing RTIs. The mechanisms driving behaviour change from the digital intervention appeared to map onto the theories of social cognitive theory and the necessity-concerns framework to change behaviour. We drew on social cognitive theory and the necessity-concerns framework to develop a digital intervention that aimed to increase nasal spray use. This qualitative study suggested that these theories underpinning the digital intervention usefully capture the key beliefs influencing nasal spray usage. Participants reported positive expectations of the use, benefits, and efficacy of the spray and higher self-efficacy encouraged greater nasal spray use and long-term use. Barriers included negative expectations of the spray efficacy and use, and low self-efficacy and skills that were accompanied by lower nasal spray usage and sub-optimal use. These findings will inform the development of future digital interventions using nasal sprays, addressing concerns such as their classification as medication, to enhance RTI prevention in high-risk populations, including those with weakened immune systems.

## Supporting information

S1 File Detailed nasal spray usage instruction. (DOCX)

S2 File Standards for reporting qualitative research (SRQR) 2014 checklist. (DOCX)

S3 File Interview topic guide. (DOCX)
