# Supplementary material for: A qualitative process evaluation of a nasal spray intervention to prevent respiratory tract infections
Source: PLoS One. 2025 Apr 29;20(4):e0321314. doi: 10.1371/journal.pone.0321314 (PMC12040087; doi:10.1371/journal.pone.0321314)
Supplement: S1 File — (DOCX) [file pone.0321314.s001.docx]

**Nasal Spray Usage Instructions**

Participants were instructed to use the nasal spray in three ways during the trial. First, a**t the first signs of infection, where they were instructed to use the nasal spray u**p to six times daily, with two sprays in each nostril, until two days after symptoms disappear. **Second, after potential exposure to infection**(e.g., public transport, supermarkets, cafes/pubs) in which they were instructed to use the nasal spray twice in each nostril immediately following exposure, then an hour later, and then last thing at night. Third, **prolonged exposure to infection**(e.g., living with or close contact with someone who has an infection) in which they were instructed to use the nasal spray up to six times a day, with two sprays in each nostril, until the close contact has recovered These usage instruction were based on manufacturer recommendations and input from the research team.
