# Supplementary material for: A qualitative process evaluation of a nasal spray intervention to prevent respiratory tract infections
Source: PLoS One. 2025 Apr 29;20(4):e0321314. doi: 10.1371/journal.pone.0321314 (PMC12040087; doi:10.1371/journal.pone.0321314)
Supplement: S3 File — (DOCX) [file pone.0321314.s003.docx]

**Interview Topic Guide**

| **Questions** |
| --- |
| **Section 1: Background** |
| 1. Can you tell me about any infections that you tend to have [repeat list of RTIs if necessary: colds, flu, coughs, chest infections, bronchitis, ear infections, sinusitis, sore throats, throat infections and tonsillitis].   Prompts:   - 1. Tell me about the different type of infections you tend to have   2. When do you tend to get your infections?   3. How often do you tend to get your infections?   4. How do your infections impact upon day-to-day life?  1. Before you started this research study, what things do you do to avoid getting these infections? |
| **Section 2: Beliefs about nasal sprays and experiences of using spray** |
| 1. Can you tell me what made you decide to sign up for this research? 2. How did you feel when you were told you would be in the group that would be using the nasal spray?    1. What did you like about being in this group?    2. What did you dislike about being in this group?    3. Did you have any concerns about being in this group? 3. You were sent a nasal spray in the post when you started this research. What did you think about receiving this? 4. [If used] How did you find using it?    1. What did you like about using it?    2. What did you dislike about using it?    3. What made it easy to use?    4. What made it difficult to use? Did you have any problems with using the spray? 5. [If didn’t use] Can you tell me what stopped you from using the spray? 6. When did you tend to use the spray?    1. What situations did you use it in?    2. What made you decide to use it on these occasions?    3. Can you talk me through how you got on with the spray that time/those times?    4. The website also suggested you could use it *[mention if they haven’t already: when they were in close contact with someone who has an infection; when they had been around lots of people who could have an infection mention; when they felt the first signs of infection]*. Did you use it in these situations?       1. If not, what stopped you from using the spray in these situations? 7. Because it was part of a research study the spray has a blank/plain label on it and was just called a ‘gel-based nasal spray’ or a ‘liquid-based nasal spray’. Can you tell me about any thoughts you had about exactly what the spray is? 8. Can you tell me about any changes you have noticed since using the spray?    1. [If changes] Why do you think these changes happened?    2. [If no changes] Why do you think you didn’t experience any changes?    3. [If not mentioned RTIs] Have you noticed any changes to how often you get infections/how bad these infections are [amend depending on their RTI experiences]? 9. Can you tell me about anything you do differently now to avoid getting infections? 10. Since being in this research study, how do you feel about infections now? 11. Do you think you will continue using the nasal spray after this research is finished?     1. Why/why not? |
| **Section 3: Experiences of Immune Defence website for nasal sprays** |
| 1. I’m really interested in hearing your views on the information and advice provided by the Immune Defence website that provides support for using the nasal spray, can you tell me what you thought about it?    1. Can you tell me about anything you liked about the information and advice in the Immune Defence website? Why is this aspect important to you?    2. Can you tell me about anything you disliked about the information and advice in the Immune Defence website? Why is this aspect important to you? 2. What did you take away from the website? 3. The website explained how we get infections and how nasal sprays might help stop infections. What did you think about this information? 4. I am also really interested in your own thoughts about how nasal sprays help stop infections. What do you think is happening when you use the nasal spray? 5. [If participant hesitates, use the following prompt] Just in your own words – don’t worry about using the scientific terms. 6. [Adapt based on answers to Q17] The website talks about how viruses get into our body and cause infections, how we don’t get ill when only a little bit of virus gets in, we only get ill when lots of virus gets in and takes hold before our bodies can fight back. What did you think about this message? 7. [Adapt based on answers to Q17] The website talks about how the nasal spray can stop infections developing or make your symptoms not as bad or as long by helping clean viruses out of our nose so that it is harder for viruses to take hold and make you ill. What did you think about this message? 8. *[If used spray]* We are curious about how the Immune Defence website and the booklet we sent in the post helps people to use nasal sprays. What was it about these resources that helped you to use the nasal spray? 9. When did you tend to log onto the website? Why these times? 10. The research study will continue for another X months. Do you think you will keep on using the Immune Defence website over this time?     1. Why/why not? 11. In the website you have the option to watch a video about how to use your nasal spray. Can you remember whether you watched this video?     1. How did you find it?     2. What did you find helpful?     3. What did you find unhelpful?     4. [If didn’t use] Can you tell me what stopped you from looking at the video? 12. A little booklet called “[iG*El Based/Liquid Based Nasal Spray- Helping You Fight Infections*]” should have arrived in the post with the spray. Can you tell me about whether you looked at this?     1. How did you find it?     2. What did you find helpful?     3. What did you find unhelpful?     4. [If didn’t look at it] Can you tell me what stopped you from reading it? 13. The website sends you emails with additional information and advice about using nasal sprays. Can you tell me about how you found the emails?     1. Can you tell me what you thought about what the emails said?     2. What did you think about how often you received the emails?   For those who have not used much of the intervention   1. We are interested to hear from people who did not use much of the Immune Defence website, can you tell me what stopped you from using the website? 2. What would have made you more likely to use Immune Defence? |
| **Section 4: COVID-19** |
| We are interested in how people may have been affected by the COVID-19 pandemic.   1. If you think back to a couple of years ago, what things were you doing at the height of the pandemic to avoid getting COVID-19?    1. Have you changed which things you’re doing now, how often, or when you are doing them?    2. Have you been doing any of these things to avoid catching infections this year? (to explore changes in perceived risk for season 3) 2. How have you found using the nasal spray alongside these things? |
| **Section 5: Trial procedures** |
| It was interesting to hear about your experiences of using the Immune Defence website and nasal sprays. Now, I’d like to hear about your experiences of the other aspects of the research.   1. Can you tell me about any problems you experienced when signing up for the research? 2. At the beginning of the research and every month since, you will have been invited to complete a questionnaire online. Can you tell me about how you found completing the questionnaires?    1. Can you tell me about any problems you came across when completing them? 3. [If they have been in the study for longer than 6 months and have received some of the follow-up texts/calls] How did you find these calls/texts? |
| **Section 6: Finish** |
| 1. Do you have anything else you would like to tell me about your experiences of the Immune Defence study, websites or using nasal sprays that we haven’t already covered? |
